# RmtC and RmtF 16S rRNA Methyltransferase in NDM-1–Producing *Pseudomonas aeruginosa*

**DOI:** 10.3201/eid2111.150271

**Published:** 2015-11

**Authors:** Mohibur Rahman, Kashi Nath Prasad, Ashutosh Pathak, Binod Kumar Pati, Avinash Singh, Cristina M. Ovejero, Saheem Ahmad, Bruno Gonzalez-Zorn

**Affiliations:** Sanjay Gandhi Postgraduate Institute of Medical Sciences, Lucknow, India (M. Rahman, K.N. Prasad, A. Pathak, B.K. Pati, A. Singh);; Integral University, Lucknow (M. Rahman, S. Ahmad);; Complutense University of Madrid, Madrid, Spain (C.M. Overjero, B. Gonzalez-Zorn)

**Keywords:** Pseudomonas aeruginosa, bacteria, methyltransferases, aminoglycosides, antimicrobial resistance, meropenem, imipenem, NDM-1, Lucknow, India

## Abstract

We investigated 16S rRNA methyltransferases in 38 *bla*_NDM-1_–positive *Pseudomonas aeruginosa* isolates and found RmtC in 3 isolates, 1 of which also harbored RmtF. The isolates were clonally unrelated; *rmtC* and *rmtF* genes were located on a chromosome with the *bla*_NDM-1_ gene. Strategies are needed to limit the spread of such isolates.

*Pseudomonas aeruginosa* causes severe and chronic invasive infections in critically ill patients. Aminoglycosides are used either alone or in combination with β-lactams as effective agents for treating such infections (*1*). Aminoglycosides block protein synthesis by binding to bacterial 16S rRNA of the 30S ribosomal subunit. Methylation of 16S rRNA makes bacteria highly resistant to aminoglycosides (*2*). Increasing instances are reported of 16S rRNA methyltransferase (16S RMTase)–producing, Gram-negative bacteria that confer high levels of resistance to aminoglycosides. Eleven types of 16S RMTases (ArmA, RmtA–RmtH, and NpmA) have so far been reported in several nosocomially transmitted pathogens, including *P. aeruginosa* (*2*–*5*). Recently, 16S RMTases have been reported in association with the New Delhi metallo-β lactamase-1 (NDM-1) in *Enterobacteriaceae* (*3*). However, such association has not been reported in *P. aeruginosa*. Therefore, we investigated the presence of 16S RMTases in NDM-1–positive *P. aeruginosa* isolates recovered from different clinical specimens.

## The Study

**Figure 1:**
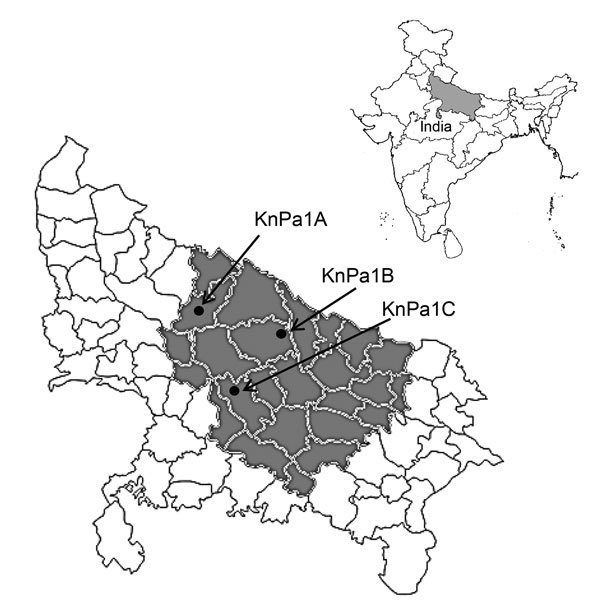
Location of Uttar Pradesh state, India, showing geographic location of patients infected with 16S rRNA methyl transferase–positive *Pseudomonas aeruginosa* (gray shading) and RmtC-positive isolates KnPa1A, KnPa1B, and KnPa1C (black dots); KnPa1B was also positive for RmtF. Inset shows location of Uttar Pradesh within India.

A total of 130 consecutive *P. aeruginosa* isolates recovered from different clinical specimens at Sanjay Gandhi Postgraduate Institute of Medical Sciences in Lucknow, Uttar Pradesh, India, during November 2013–April 2014 were included in the study; all specimens were collected from within the state (Figure 1). *P. aeruginosa* isolates were identified by standard microbiological techniques (*6*) and further confirmed by Phoenix automated identification and sensitivity systems (BD Biosciences, San Jose, CA, USA). The drug susceptibility profile was interpreted by using Clinical and Laboratory Standards Institute breakpoints (*7*). A total of 38 (29.23%) isolates were resistant to meropenem and imipenem. These isolates were subjected to PCR by using *bla*_NDM_­­­­–specific primers (*8*) followed by amplicon sequencing. Sequencing identified *bla*_NDM-1_ in all 38 isolates. The isolates were further screened for high-level aminoglycoside resistance by their ability to grow on Muller Hinton agar containing amikacin and gentamicin 256 mg/L each as a marker for 16S RMTase (*3*). A total of 33 (86.84%) isolates were positive for high-level aminoglycoside resistance. Geographic locations of patients infected with these isolates are provided in Figure 1. Each of these isolates were further subjected to PCR for detection of 16S RMTases (ArmA and RmtA–RmtH) by using primers and conditions described previously (*2*–*5*); 17 (51%) isolates were positive for 16S RMTases. Their distributions were as follows: ArmA in 6 (18%); RmtB in 4 (12%); ArmA + RmtB in 4 (12%); RmtC in 2 (KnPa1A and KnPa1C) (6%); and RmtC + RmtF in 1 (KnPa1B) (3%). RmtC and RmtF had not previously been reported in *P. aeruginosa*. KnPa1A, KnPa1B, and KnPa1C were further characterized; sequence analysis of amplicons confirmed *rmtC* with 100% nucleotide identity originally described in *Proteus mirabilis* strain ARS68 from Japan (*9*) and assigned EMBL/GenBank nucleotide accession nos. KJ476816, KJ476817, and KJ476818. MICs of the 3 isolates for different aminoglycosides, β-lactams, β-lactam/β-lactamase inhibitor combinations, carbapenems, and colistin are provided in the Table.

KnPa1A was isolated from surgical drainage from a woman, 59 years of age, who had hypertension and underwent an abdominal hysterectomy for cervical carcinoma, followed by external beam radiotherapy. Eventually, a vesicovaginal fistula developed, and pelvic fluid was collected. *P. aeruginosa* (KnPa1A) was isolated from pelvic drainage. Her condition stabilized, and she was discharged with advice for repair of the fistula, but she did not return for further treatment. During hospitalization, she received multiple antimicrobial drugs.

KnPa1B was isolated from endoscopic nasobiliary drainage (ENBD) collected from a man, 57 years of age, who had extrahepatic biliary obstruction as a complication of hilar cholangiocarcinoma. Endoscopic retrograde cholangiopancreatography with stenting was performed. Stent block and fever occurred, necessitating a repeat of the procedure and drainage of fluid. He was discharged with the drainage tube in situ and was advised to return for surgery. *P. aeruginosa* (KnPa1B) was isolated from ENBD. The patient did not report for follow-up treatment.

KnPa1C was isolated from a man, 68 years of age, who had diabetes mellitus and hypertension. He had stricture of the urethra and meatal narrowing after having a transurethral prostate resection. He underwent urethral dilatations and placement of a urinary catheter. A urinary tract infection was diagnosed, and *P. aeruginosa* (KnPa1C) was recovered from urine. The patient received piperacillin/tazobactam and colistin combination therapy; urine culture was sterile on day 3 posttreatment.

**Figure 2:**
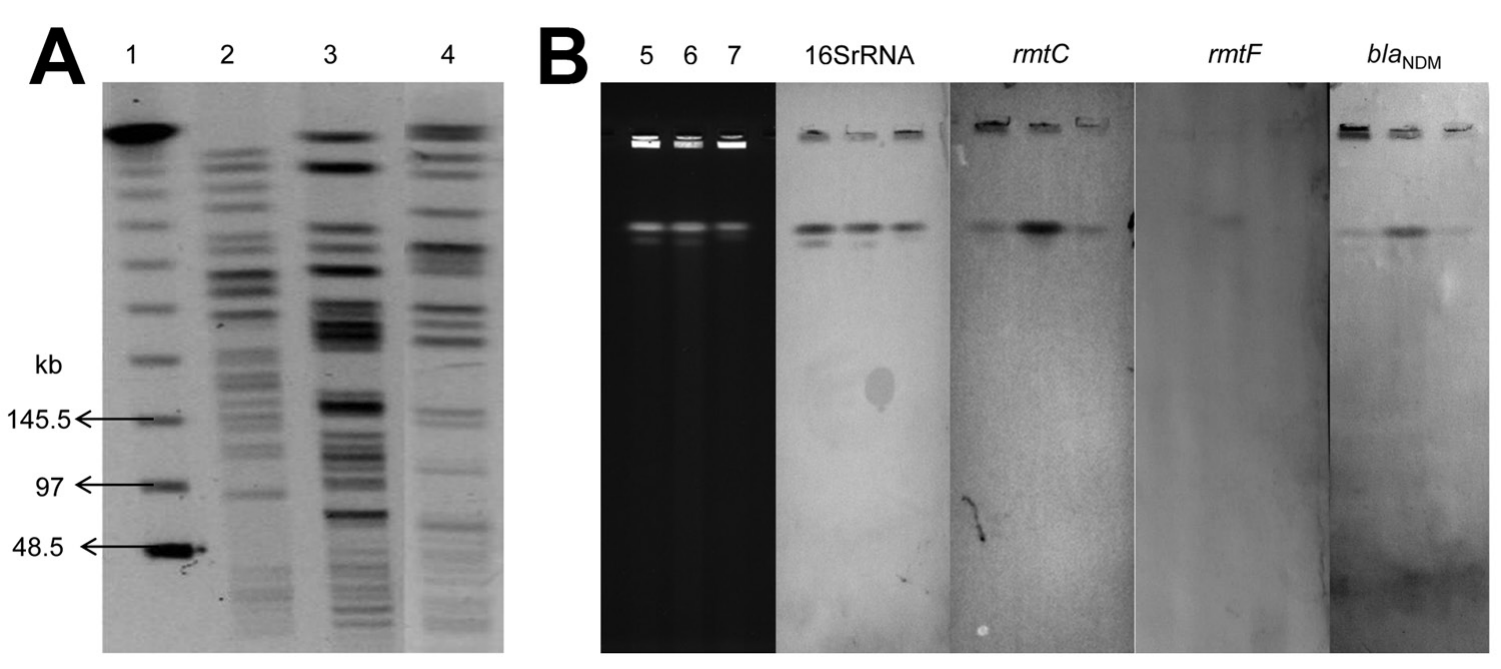
A) Pulsed-field gel electrophoresis patterns of *rmtC*-positive *Pseudomonas aeruginosa*. Lane 1, λ ladder; 2, KnPa1A; 3, KnPa1B; 4, KnPa1C. B) Chromosomal location of *rmtC, rmtF*, and *bla*_NDM-1_ genes by I-CeuI-digested genomic DNA of *P. aeruginosa* isolates. Lane 5, KnPa1A; 6, KnPa1B; 7, KnPa1C; smears show Southern blot analysis of genomic DNA with probes specific to 16S rRNA, RmtC, RmtF, and NDM-1 genes.

Resistance genes such as metallo-β-lactamases (e.g., IMP, VIM, SIM, GIM, SPM) and extended-spectrum β-lactamases such as TEM, SHV, CTX-M, and AmpC were detected by using PCR (*8*) (Table). To study genetic relatedness among the 3 isolates, genomic DNA in agarose blocks was separated on 1.0% agarose gels in 0.5 × tris-borate-EDTA buffer with the CHEF II D-Mapper XA pulsed-field gel electrophoresis system (Bio-Rad, Hercules, CA, USA) following standard conditions (*10*). All 3 isolates had different PFGE patterns (Figure 2). Multilocus sequence typing (MLST) was done according to protocols described in the *Pseudomonas* *aeruginosa* MLST Database (http://pubmlst.org/paeruginosa). Seven chromosomal genes were PCR amplified and sequenced; the sequences were compared with those on the MLST database to determine allele numbers and sequence types (STs). KnPa1A, KnPa1B, and KnPa1C belonged to ST764, ST902, and ST880, respectively.

**Table:** MICs of different antimicrobial drugs, resistance genes, and association of IS*Ecp1* with *rmtC* in 3 *Pseudomonas aeruginosa* clinical isolates, India, 2013–2014*

Isolate	MIC, mg/L											Antimicrobial drug resistance genes	Association of IS*Ecp1* with *rmtC*
	CAZ	CTX	FEP	ATM	CPS	TZP	IPM	MER	COL	AK	G		
KnPa1A	>512	>512	512	8	256	64	16	32	2	>512	>512	*bla*_NDM-1_, *rmtC*	Intact
KnPa1B	256	>512	>512	16	128	32	16	64	2	>512	>512	*bla*_NDM-1_, *rmtC,rmtF, bla*_CMY-2_	Truncated
KnPa1C	>512	>512	512	16	128	64	32	128	2	>512	>512	*bla*_NDM-1_, *rmtC*, *bla*_TEM-1_, *bla*_CTX-M-15_	Truncated

*CAZ, ceftazidime; CTX, cefotaxime; FEP, cefepime; ATM, aztreonam; CPS, cefoperazone/sulbactam; TZP, piperacillin/tazobactam; IPM, imipenem; MER, meropenem; COL, colistin; AK, amikacin; G, gentamicin.

*ISEcp*1 was previously shown to promote both expression and transposition of *rmtC* (*11*); hence, to assess association of *ISEcp*1 with *rmtC*, PCR was performed on the 3 isolates with primer pairs ISEcpIR-F and *rmtC*-down and *ISEcp*1–5′ and RMTC-R, as described (*12*). Sequence analysis of amplicons revealed association of an intact IS*Ecp*1 element with *rmtC* in KnPa1A; however, complete *ISEcp*1 could not be amplified in KnPa1B and KnPa1C, corroborating earlier observations of either partial deletion of this element or role of a different IS*Ecp1*-like element in the spread of *rmtC* in gram-negative bacteria (*13*). Attempts to transfer *rmtC* from all 3 isolates to rifampin-resistant *Escherichia coli* 20R764 and ciprofloxacin-resistant *P. aeruginosa* of strain 105 through conjugation were unsuccessful (*1*,*13*). Repeated attempts to obtain amikacin-resistant (MIC ≥16 g/mL) transformants of *E*. *coli* DH5α and *P*. *aeruginosa* PA01 by electroporation with plasmid preparation by using the Kado and Liu method (*14*) were also unsuccessful, despite successful transfer of control plasmids. To determine the location of *rmtC, rmtF*, and *bla*_NDM-1_, genomic DNA from the 3 isolates was digested separately with restriction enzyme I-Ceu-1 (New England Biolabs, Beverly, MA, USA), separated by PFGE, and subsequently assayed with 16S rRNA, *rmtC, rmtF*, and *bla*_NDM-1_ probes (*13*). All these probes were hybridized with chromosomal DNA (Figure 2) and not with plasmid extract. This result shows that *rmtC, rmtF*, and *bla*_NDM-1_ were located and stabilized on the chromosome of *P. aeruginosa*.

## Conclusions

We describe an occurrence of 16S RMTases RmtC and RmtF in clinical isolates of *P. aeruginosa* co-producing *bla*_NDM-1_. The *rmtC* and *rmtF* genes might have been acquired from plasmids as part of mobile genetic elements and finally integrated and stabilized on the chromosome, but the underlying mechanism of transmission needs to be elucidated. Further, spread of multidrug-resistant *P. aeruginosa* strains expressing RmtC with and without an intact IS*Ecp1* element and NDM-1 is of major clinical concern and calls for further studies to limit the spread of such strains.
